# Exploration of comorbidity mechanisms between chronic pain and depression: Machine learning prediction models and SHAP interpretability analysis based on the CHARLS cohort

**DOI:** 10.1371/journal.pone.0349135

**Published:** 2026-06-08

**Authors:** Tao-Ming Dai, Jie Yuan, Yue-Yang Ma, Jun-Jun Liu

**Affiliations:** 1 Affiliated Hospital 6 of Nantong University, Yancheng, China; 2 Yancheng Third People’s Hospital, the affiliated hospital of Jiangsu Medical College, Yancheng, China; 3 Yancheng Vocational College of Industry, Yancheng, China; National University of Science & Technology, PAKISTAN

## Abstract

**Introduction:**

With rapid population aging in China, depression among middle-aged and older adults has become a major public health concern. Chronic pain and sociodemographic factors are closely associated with depressive symptoms, yet their combined and heterogeneous effects are difficult to capture using traditional analytical approaches. Interpretable machine learning provides a framework to explore depression-related risk patterns within a biopsychosocial perspective.

**Methods:**

Data were obtained from seven waves of the China Health and Retirement Longitudinal Study (CHARLS, 2011–2020), including 38,970 adults aged 45 years and older. Depressive symptoms were assessed using the 10-item Center for Epidemiologic Studies Depression Scale (CES-D-10). Predictors covering sociodemographic characteristics, lifestyle factors, and pain at specific anatomical sites were selected using LASSO regression and recursive feature elimination. Seven machine learning models—logistic regression, Bernoulli and Gaussian Naive Bayes, support vector machine (SVM), random forest, extreme gradient boosting (XGBoost), and k-nearest neighbors—were developed and evaluated using accuracy, area under the receiver operating characteristic curve (AUC), precision, recall, and F1-score. Model interpretability was assessed using SHapley Additive exPlanations (SHAP).

**Results:**

Chronic pain, particularly involving the lower limbs and spine, showed a strong association with depressive status. Model accuracy ranged from 61.6% to 71.9%, with AUC values between 0.61 and 0.72. SVM and Gaussian Naive Bayes demonstrated relatively better overall discrimination, though sensitivity for depressive cases remained limited. SHAP analysis identified lower limb pain and a nonlinear association with body mass index as key contributors to depression risk prediction.

**Discussion:**

Integrating interpretable machine learning with a biopsychosocial framework highlights the dominant role of pain-related features in depression risk patterns among older adults. Given limited sensitivity for depressive case identification, these models are more suitable for population-level risk exploration than direct clinical screening.

## Introduction

As China undergoes rapid population aging, mental health issues among middle-aged and older adults have become a critical public health concern. According to the World Health Organization, the prevalence of depressive disorders among individuals aged 60 and above reaches 13.5%, with comorbid physical illnesses substantially increasing the risk of functional impairment and disability. Recent findings from the China Health and Retirement Longitudinal Study (CHARLS) demonstrate a persistent upward trend in the detection rate of depressive symptoms within this demographic, underscoring the urgent need for targeted prevention strategies. However, the multifactorial etiology of depression—shaped by the dynamic interplay of biological, psychological, and social determinants—poses significant challenges for traditional analytical approaches.

To date, most research has focused on isolated risk factors [1–6], with limited attention to the complex interdependencies across systems. Sociodemographic studies have identified female sex, lower educational attainment, and other factors as significant correlates of depression, yet these variables fall short in explaining the heterogeneity of depressive outcomes among individuals facing similar social stressors. In the field of neurobiology, there is growing evidence that chronic pain and depression share common neurotransmitter pathways [7], yet few studies have systematically examined how pain in distinct anatomical regions differentially contributes to depressive symptomatology. Additionally, existing predictive models largely rely on linear regression and other conventional statistical techniques [1–10], which are often inadequate for capturing nonlinear, high-dimensional interactions, and offer limited interpretability in clinical contexts.

This study addresses these critical gaps by integrating the biopsychosocial medical framework with contemporary machine learning methodologies. Specifically, it aims to advance the field in three key areas. First, we incorporate 14 anatomically specific pain symptoms to enrich the phenotypic characterization of the bidirectional “pain–depression” relationship. Second, we implement a hybrid feature selection strategy—combining LASSO regression and recursive feature elimination (RFE)—to construct a parsimonious yet clinically meaningful set of predictors. Third, we employ SHAP (SHapley Additive exPlanations), an interpretable artificial intelligence technique, to elucidate the dynamic interactions between social determinants and somatic symptoms in the prediction of depression risk.

Leveraging a nationally representative, decade-long longitudinal dataset from CHARLS, this research offers methodological innovations and empirical insights to support the development of a culturally and contextually tailored depression risk early warning system for China’s aging population.

## Materials and methods

### Data source

This study utilized publicly available data from the China Health and Retirement Longitudinal Study (CHARLS), covering the period from 2011 to 2020. CHARLS is a nationally representative, population-based longitudinal survey designed to collect high-quality microdata on individuals and households aged 45 years and older in China. The project aims to support interdisciplinary research on population aging and inform evidence-based policy. The dataset is publicly available and fully anonymized, and can be accessed from the official CHARLS website (http://charls.pku.edu.cn/en). The original CHARLS study was approved by the Institutional Review Board of Peking University (IRB00001052-11015), and all participants provided informed consent at the time of data collection. The current analysis is based on secondary use of de-identified data and does not involve any new data collection or direct contact with human subjects; thus, no additional ethical approval was required.

Depressive symptoms were measured using the short-form 10-item Center for Epidemiologic Studies Depression Scale (CES-D-10), with each item scored from 0 to 3 (0 = rarely or none of the time; 3 = most or all of the time). The scale captures four core dimensions: depressive affect (e.g., sadness, hopelessness), positive affect (e.g., reduced enjoyment), somatic symptoms (e.g., appetite loss), and cognitive/behavioral symptoms (e.g., poor concentration, reduced activity). The CHARLS research team at Peking University has validated this scale in Chinese populations and recommends using the total score as a screening index for depressive symptoms in individuals aged 45 and above. International studies such as the U.S. Health and Retirement Study (HRS) and the Survey of Health, Ageing and Retirement in Europe (SHARE) also employ the CES-D-10 for depression screening, with its reliability and validity widely recognized in epidemiological research.

### Selection of predictor variables

Based on the biopsychosocial model and the theory of chronic disease comorbidity, this study constructed a multidimensional analytic framework to identify factors associated with depressive states. Sociodemographic factors: Gender, age, marital status, educational attainment, and place of residence were included as key indicators of social resource access and mental health inequality. Gender differences may reflect hormonal or role-based stress; age groups (<60 and ≥60) were used to capture physiological and social role transitions across the life course. Marital status served as a proxy for social support, while education reflects socioeconomic status and health literacy. Urban versus rural residence was considered to reflect disparities in healthcare access and environmental stress.

Lifestyle factors: Smoking status, alcohol consumption, and body mass index (BMI) were included as proxies for health behaviors. Smoking and alcohol use may influence emotional states via neurotransmitter regulation or stress coping mechanisms. BMI was categorized as <18.5, 18.5–23.9, and >23.9 to represent undernutrition, normal metabolism, and metabolic dysregulation, respectively—all of which have been linked to inflammation and depression risk.

Somatic symptom factors: Fourteen specific anatomical pain locations (e.g., headache, low back pain, knee pain) were systematically included. Based on the bidirectional “pain–depression” hypothesis, pain is considered both a biomarker of central sensitization and a potential psychological stressor due to activity limitations. The indicators encompass central (e.g., headache), musculoskeletal (e.g., back pain), and peripheral (e.g., toe pain) pain types to explore heterogeneity in symptom effects. Detailed descriptions and coding of all CHARLS variables used in this study are provided in S1 File.

All variables were initially validated using univariate analysis to confirm significant associations with depressive states (*P* < 0.001), ensuring consistency with the exposure–outcome temporal logic required in clinical epidemiology. The final indicator system integrates classical social determinants with cross-system chronic pain factors, offering an empirical basis to explore the multilayered drivers of depression.

### Analytical methods

**Fig 1:**
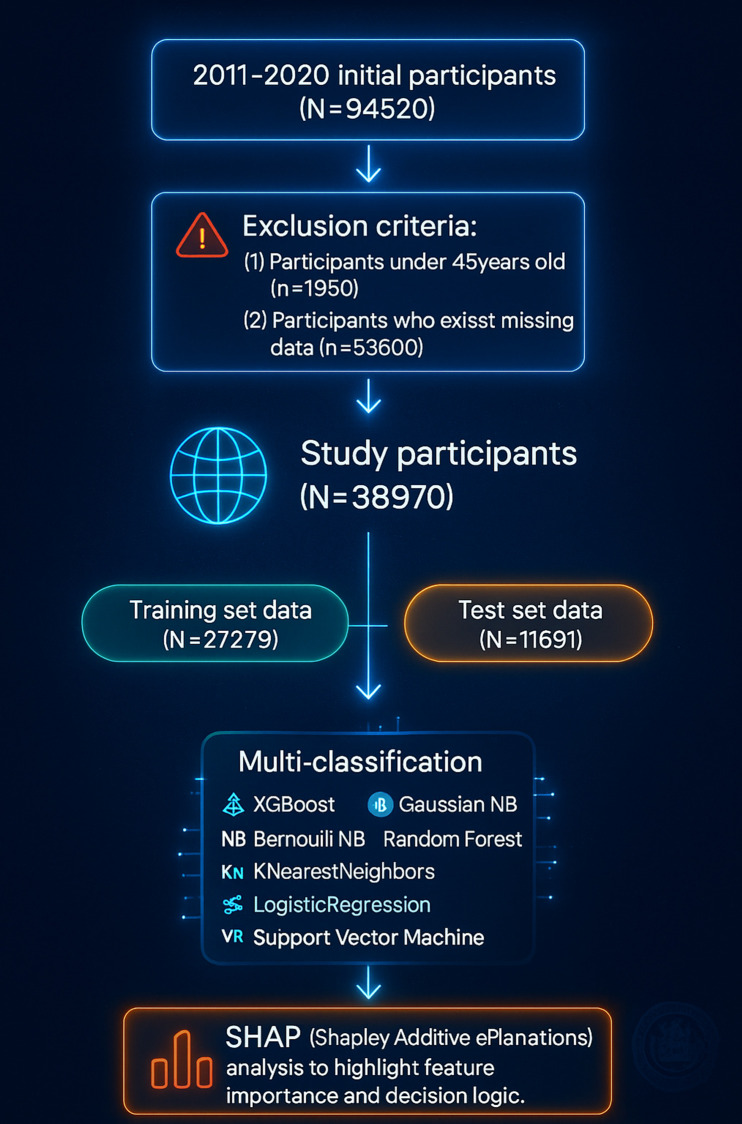
A flowchart describing the general framework of the study.

This study constructed a retrospective cohort based on seven waves of longitudinal data from the China Health and Retirement Longitudinal Study (CHARLS) collected between 2011 and 2020, and employed a multi-stage modeling strategy to systematically examine factors associated with depressive status and their predictive contributions. The overall analytical workflow of the study is illustrated in Fig 1. To optimize predictor selection, candidate variables were first screened using univariate analyses (*P* < 0.001), followed by a dual-validation feature selection approach combining L1-regularized regression (LASSO, α = 0.01) and recursive feature elimination (RFE; base learner: random forest with n_estimators = 200). Ultimately, eight consensus features consistently retained across methods were selected, including education level, body mass index (BMI), and six pain-related symptoms (e.g., lower limb pain and low back pain).

Based on the selected feature set, six categories of machine learning models were constructed for comparison, including a linear model (logistic regression with L2 regularization), probabilistic models (Gaussian and Bernoulli naïve Bayes), a support vector machine with a radial basis function kernel, ensemble learning models (random forest and extreme gradient boosting), and a distance-based model (k-nearest neighbors). To reduce manual tuning bias and improve model generalizability, key hyperparameters for all models were optimized using Bayesian optimization. Specifically, internal validation was performed within the training set using a stratified sampling framework that preserved the class distribution of depressive status, and performance metrics relevant to class imbalance (such as the area under the receiver operating characteristic curve, recall, or F1-score) were used as objective functions. For each model, 50 optimization iterations were conducted, with the hyperparameter search space covering regularization strength (e.g., the C parameter in logistic regression), kernel-related parameters (e.g., C and γ in the support vector machine), model complexity and ensemble size for tree-based models (e.g., n_estimators, max_depth, min_samples_split, and min_samples_leaf for random forest; and max_depth, learning_rate, subsample, colsample_bytree, and n_estimators for XGBoost), as well as the number of neighbors (k) and distance metrics for the k-nearest neighbors model. The optimal hyperparameter configurations identified through Bayesian optimization were used to refit each model on the full training set, and final model performance was evaluated on an independent test set generated using a stratified 7:3 train–test split. An illustrative version of the main analytical workflow is provided in S2 File to enhance transparency of the modeling process.

## Results

### Baseline characteristics and univariate analysis

This study included a total of 38,970 participants. The demographic characteristics and univariate analysis results of the study population are summarized in Table 1. The demographic characteristics of the sample were as follows: females accounted for 56.27% (*n* = 21,924), while males comprised 43.73% (*n* = 17,046). Regarding age, 53.21% (*n* = 20,734) of participants were younger than 60 years, and 46.79% (*n* = 18,236) were aged 60 years or above, indicating a population primarily consisting of middle-aged and older adults. In terms of marital status, the majority of participants were living with a spouse or partner (87.40%, *n* = 34,059), while 12.60% (*n* = 4,911) reported having no spouse or partner. Educational attainment demonstrated a clear downward trend: 45.14% (*n* = 17,593) had less than primary education, 24.91% (*n* = 9,709) had completed primary school, 19.56% (*n* = 7,622) had junior high school education, and only 10.38% (*n* = 4,046) had attained high school or above, reflecting generally low educational levels in the study population. With regard to place of residence, 62.64% (*n* = 24,420) lived in urban areas, while 37.36% (*n* = 14,550) resided in rural areas. Overall, the study population was characterized by a predominance of middle-aged and elderly individuals, a higher proportion of women, low levels of education, and urban residency. Univariate analysis revealed statistically significant associations between depressive symptoms and all demographic characteristics as well as somatic pain symptoms (all *P* < 0.001).

**Table 1:** Univariate analysis of demographic characteristics and somatic pain symptoms.

Variable	*n*	Non-depressed (*n*, %)	Depressed (*n*, %)	*P*-value
Sex				0.000***
Female	21,924	13,055 (59.55%)	8,869 (40.45%)	
Male	17,046	12,416 (72.84%)	4,630 (27.16%)	
Age				0.000***
< 60	20,734	13,997 (67.51%)	6,737 (32.49%)	
≥ 60	18,236	11,474 (62.92%)	6,762 (37.08%)	
Marital status				0.000***
With spouse/partner	34,059	22,825 (67.02%)	11,234 (32.98%)	
Without spouse/partner	4,911	2,646 (53.88%)	2,265 (46.12%)	
Education level				0.000***
< Primary	17,593	10,082 (57.31%)	7,511 (42.69%)	
Primary	9,709	6,573 (67.70%)	3,136 (32.30%)	
Junior high	7,622	5,568 (73.05%)	2,054 (26.95%)	
High school and above	4,046	3,248 (80.28%)	798 (19.72%)	
Residence				0.000***
Rural	14,550	10,420 (71.62%)	4,130 (28.38%)	
Urban	24,420	15,051 (61.63%)	9,369 (38.37%)	
Alcohol consumption				0.000***
No	26,178	16,348 (62.45%)	9,830 (37.55%)	
Yes	12,792	9,123 (71.32%)	3,669 (28.68%)	
Smoking				0.000***
No	28,718	18,358 (63.93%)	10,360 (36.08%)	
Yes	10,252	7,113 (69.38%)	3,139 (30.62%)	
BMI				0.000***
< 18.5	2,283	1,257 (55.06%)	1,026 (44.94%)	
18.5–23.9	18,837	12,046 (63.95%)	6,791 (36.05%)	
> 23.9	17,850	12,168 (68.17%)	5,682 (31.83%)	
Headache				0.000***
No	33,806	23,881 (70.64%)	9,925 (29.36%)	
Yes	5,164	1,590 (30.79%)	3,574 (69.21%)	
Shoulder pain				0.000***
No	34,026	23,770 (69.86%)	10,256 (30.14%)	
Yes	4,944	1,701 (34.41%)	3,243 (65.60%)	
Wrist pain				0.000***
No	36,331	24,717 (68.03%)	11,614 (31.97%)	
Yes	2,639	754 (28.57%)	1,885 (71.43%)	
Finger pain				0.000***
No	36,303	24,697 (68.03%)	11,606 (31.97%)	
Yes	2,667	774 (29.02%)	1,893 (70.98%)	
Chest pain				0.000***
No	36,599	24,822 (67.82%)	11,777 (32.18%)	
Yes	2,371	649 (27.37%)	1,722 (72.63%)	
Stomach pain				0.000***
No	35,723	24,453 (68.45%)	11,270 (31.55%)	
Yes	3,247	1,018 (31.35%)	2,229 (68.65%)	
Back pain				0.000***
No	35,191	24,271 (68.97%)	10,920 (31.03%)	
Yes	3,779	1,200 (31.75%)	2,579 (68.25%)	
Low back pain				0.000***
No	31,373	22,593 (72.01%)	8,780 (27.99%)	
Yes	7,597	2,878 (37.88%)	4,719 (62.12%)	
Hip pain				0.000***
No	37,258	24,980 (67.05%)	12,278 (32.95%)	
Yes	1,712	491 (28.68%)	1,221 (71.32%)	
Leg pain				0.000***
No	33,322	23,580 (70.76%)	9,742 (29.24%)	
Yes	5,648	1,891 (33.48%)	3,757 (66.52%)	
Knee pain				0.000***
No	33,661	23,639 (70.23%)	10,022 (29.77%)	
Yes	5,309	1,832 (34.51%)	3,477 (65.49%)	
Ankle pain				0.000***
No	36,442	24,695 (67.77%)	11,747 (32.24%)	
Yes	2,528	776 (30.70%)	1,752 (69.30%)	
Toe pain				0.000***
No	37,434	25,042 (66.90%)	12,392 (33.10%)	
Yes	1,536	429 (27.93%)	1,107 (72.07%)	
Arm pain				0.000***
No	35,054	24,256 (69.20%)	10,798 (30.80%)	
Yes	3,916	1,215 (31.03%)	2,701 (68.97%)	
Neck pain				0.000***
No	35,987	24,535 (68.18%)	11,452 (31.82%)	
Yes	2,983	936 (31.38%)	2,047 (68.62%)	

### Development of machine learning models

#### Feature selection based on Lasso and RFE.

**Fig 2:**
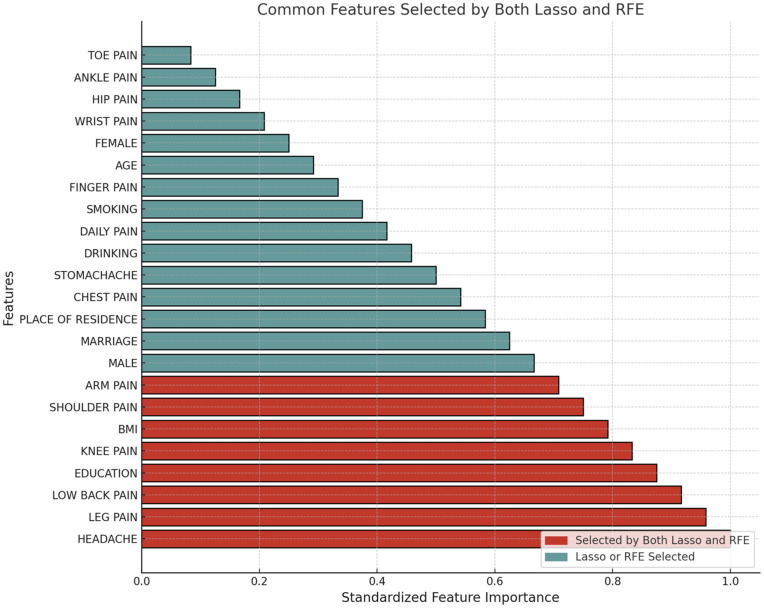
Lasso and RFE feature importance comparison.

In this study, feature selection was conducted using both Lasso regression and recursive feature elimination (RFE), which demonstrated a notable synergy in biomedical variable identification. The comparison of feature importance and the overlap between the two methods are presented in Fig 2. The Lasso algorithm selected 17 key features from demographic indicators and somatic pain symptoms, including sex (MALE), education level (EDUCATION), body mass index (BMI), and 12 pain-related variables (e.g., HEADACHE, LOW BACK PAIN). These results highlight the joint predictive value of sociodemographic characteristics and multi-site pain symptoms.

In contrast, the RFE method identified a more parsimonious subset of 10 features, among which age (AGE) and sex (FEMALE) emerged as distinct variables not selected by Lasso, providing a complementary perspective. Notably, both methods reached consensus on eight core features, including lower limb pain (LEG PAIN), upper limb pain (ARM PAIN), and shoulder pain (SHOULDER PAIN), as well as fundamental health indicators such as education level and BMI. This shared feature set constitutes a core biomarker combination for disease risk prediction.

#### Model performance evaluation and clinical applicability assessment.

**Fig 3:**
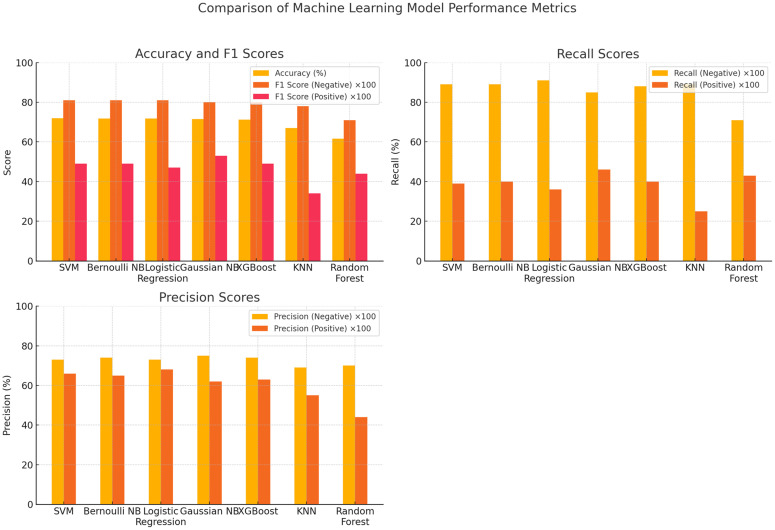
Performance metrics of machine learning models.

**Fig 4:**
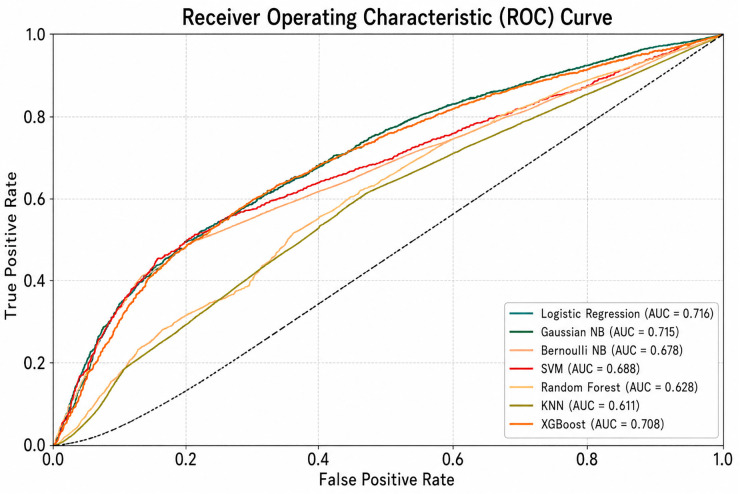
ROC curves of the machine learning models.

Seven machine learning models were evaluated for their predictive performance in identifying depressive status: Support Vector Machine (SVM), Logistic Regression, Bernoulli Naive Bayes, Gaussian Naive Bayes, K-Nearest Neighbors (KNN), Random Forest, and XGBoost. A detailed comparison of model performance across key classification metrics is presented in Table 2, while the overall distribution of performance metrics across models is visualized in Fig 3. The models were compared across key classification metrics, including accuracy, precision, recall, and F1 score, revealing notable variations in performance. In terms of overall accuracy, SVM (71.92%), Bernoulli Naive Bayes (71.87%), and Logistic Regression (71.80%) were the top performers, demonstrating stable and reliable classification abilities. These were followed by Gaussian Naive Bayes (71.47%) and XGBoost (71.28%). In contrast, KNN achieved a lower accuracy of 66.96%, while Random Forest exhibited the poorest performance with an accuracy of only 61.58%. For negative class (non-depression) classification, SVM, Logistic Regression, and both Naive Bayes models achieved high recall values (≥0.85) and F1 scores (≥0.80), with Logistic Regression achieving the highest recall at 0.91. These results indicate the strong sensitivity and robustness of these models in identifying non-depressed individuals. However, the performance for the positive class (depression cases) was generally weaker. The highest F1 score observed was 0.53 with Gaussian Naive Bayes, followed by SVM and Bernoulli Naive Bayes at 0.49. These findings suggest that current models have limited capability in detecting individuals at high risk of depression. Notably, although Logistic Regression demonstrated strong performance in detecting non-depression cases, its recall (0.36) and F1 score (0.47) for the positive class were relatively low, indicating a conservative bias in classification. In contrast, Gaussian Naive Bayes provided a better balance, with a recall of 0.46 and an F1 score of 0.53 for the positive class, highlighting its potential applicability in early depression risk screening and intervention. To further evaluate model discrimination ability, receiver operating characteristic (ROC) curve analysis was conducted. The ROC curves of the seven machine learning models are shown in Fig 4, with area under the curve (AUC) values ranging from 0.61 to 0.72. SVM and Gaussian Naive Bayes demonstrated comparatively higher AUC values, indicating better overall discrimination performance, whereas the remaining models showed moderate but comparable discriminative ability.

**Table 2:** Comparison of predictive performance metrics of machine learning models.

Model	Accuracy	AUC	Precision (Negative)	Recall (Negative)	F1 score (Negative)	Precision (Positive)	Recall (Positive)	F1 score (Positive)
Support Vector Machine (SVM)	71.92%	0.688	0.73	0.89	0.81	0.66	0.39	0.49
Bernoulli Naive Bayes	71.87%	0.678	0.74	0.89	0.81	0.65	0.4	0.49
Logistic Regression	71.80%	0.716	0.73	0.91	0.81	0.68	0.36	0.47
Gaussian Naive Bayes	71.47%	0.715	0.75	0.85	0.8	0.62	0.46	0.53
XGBoost	71.28%	0.708	0.74	0.88	0.8	0.63	0.4	0.49
K-Nearest Neighbors (KNN)	66.96%	0.611	0.69	0.89	0.78	0.55	0.25	0.34
Random Forest	61.58%	0.628	0.7	0.71	0.71	0.44	0.43	0.44

#### XGBoost-SHAP interpretable model.

**Fig 5:**
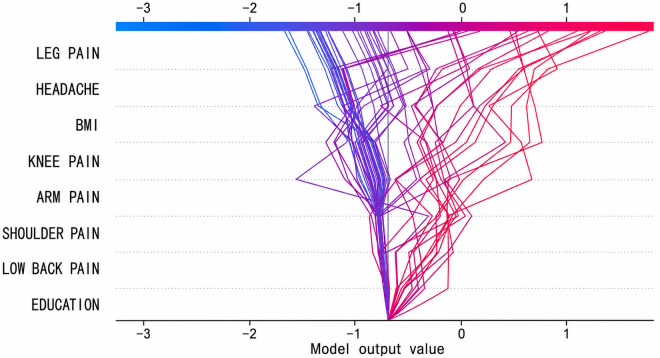
SHAP decision paths (top 50 samples).

This study employed SHAP (SHapley Additive exPlanations) analysis to elucidate the relative predictive contributions of key features and to enhance the interpretability of the machine learning models (Fig 5). By decomposing model predictions into feature-level Shapley values, the analysis provided a transparent description of how individual variables contributed to depression-related risk prediction.

Overall, pain-related symptoms exhibited stronger and more consistent predictive contributions than most sociodemographic variables, indicating their central role within the predictive framework. Among these, leg pain (LEG PAIN) showed a left-skewed SHAP value distribution, with values ranging from approximately –3–0, suggesting that the presence of leg pain was associated with an increased predicted probability of depressive status. The dense concentration of SHAP values in the negative range reflects a relatively stable contribution of this feature across individuals, highlighting its importance as a predictive marker rather than implying diagnostic specificity.

Body mass index (BMI) demonstrated a nonlinear, biphasic pattern in SHAP values. Higher BMI levels were associated with positive SHAP values (approximately +0.5 to +1.2), indicating increased predicted risk, whereas lower BMI values were associated with negative SHAP values (approximately –0.3 to –0.8). This pattern suggests a U-shaped predictive association between BMI and depressive status within the model, consistent with prior epidemiological observations, but should be interpreted as a predictive relationship rather than evidence of physiological regulation.

In contrast, education level (EDUCATION) showed a comparatively modest predictive contribution. Although higher education levels were generally associated with negative SHAP values (median approximately –0.6), indicating a directionally protective association, the overall magnitude and dispersion of SHAP values were smaller than those observed for pain-related variables. This finding suggests that education may play a secondary role in the multivariable prediction of depressive status, and its influence should be interpreted in the context of stronger somatic symptom predictors rather than as a dominant protective factor.

From a feature interaction perspective, overlapping SHAP decision paths were observed between arm pain and shoulder pain. Rather than implying a shared neurobiological mechanism, this overlap likely reflects collinearity or co-occurrence of anatomically adjacent pain symptoms within the dataset, indicating that these features may convey partially redundant predictive information in the model.

**Fig 6:**
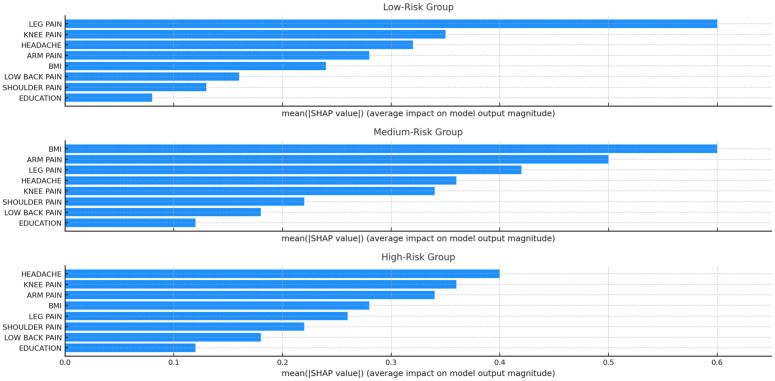
SHAP feature importance.

This study applied SHAP value analysis to compare the relative importance of key predictors—including pain locations, body mass index (BMI), and education level—across different depression risk strata. The risk-stratified SHAP feature importance patterns are illustrated in Fig 6. In the low-risk group, pain-related features such as lower limb pain (leg pain and knee pain) and headache exhibited the highest SHAP contributions, suggesting that at lower risk levels, depressive vulnerability may be primarily reflected through localized somatic symptom patterns.

In the moderate-risk group, BMI emerged as the most influential predictor, indicating that overall physical health status played a more prominent role at this stage. In addition, arm pain, leg pain, and headache continued to demonstrate meaningful predictive contributions, reflecting the coexistence of multiple somatic symptoms in individuals with intermediate depression risk.

Within the high-risk group, headache and knee pain showed substantially higher SHAP values than other variables, highlighting their dominant predictive contributions. Rather than implying causal mechanisms, this pattern suggests that specific chronic pain symptoms tend to co-occur with elevated depression risk and may serve as salient markers within high-risk populations.

Across all risk strata, education level consistently displayed relatively low SHAP contributions, indicating a limited direct role in depression prediction within the multivariable modeling framework. Overall, these findings underscore the heterogeneity of predictive feature importance across risk levels and illustrate the value of risk-stratified SHAP analysis in providing an intuitive and complementary summary to graphical explanations.

## Discussion

Using ten years of nationally representative data from the China Health and Retirement Longitudinal Study (CHARLS), this study applied interpretable machine learning approaches to systematically examine factors associated with depressive status among middle-aged and older adults. Grounded in the biopsychosocial framework, the analysis focused on identifying stable predictive associations rather than inferring causal mechanisms. By integrating multiple modeling strategies with SHAP-based interpretation, this study advances understanding of how sociodemographic characteristics and pain-related symptoms jointly shape depression-related risk patterns in later life.

The findings indicate that both sociodemographic factors and somatic pain symptoms contribute to the prediction of depressive status, which is consistent with existing evidence on the co-occurrence of chronic physical conditions and mental health outcomes [11]. Female participants exhibited a higher probability of depressive status than males, a pattern widely observed in population-based studies and potentially reflecting a combination of biological vulnerability and gender-related social stressors [12–13]. Higher educational attainment was inversely associated with depression risk, suggesting that accumulated social and cognitive resources may be linked to better psychological resilience. However, SHAP-based analyses showed that the relative predictive contribution of education was modest compared with several pain-related variables, underscoring the importance of interpreting social determinants within a multivariable predictive context rather than as dominant protective factors.

Notably, pain-related symptoms demonstrated stronger and more consistent predictive contributions than most traditional sociodemographic variables. Symptoms involving the spine and lower extremities, such as low back pain and leg pain, emerged as particularly influential predictors across models. These results align with prior research reporting close associations between chronic pain and depressive symptoms [14,15]. Rather than implying specific biological mechanisms, the observed patterns may reflect the cumulative burden of pain, functional limitation, and reduced mobility, and may provide empirical clues for future investigations into the pathways linking chronic pain and depression.

An important contribution of this study lies in highlighting heterogeneity in the predictive contributions of pain across different anatomical regions. Pain affecting the spine and lower extremities showed relatively stronger associations with depressive status than pain in other regions. This heterogeneity likely reflects differences in functional impairment, daily activity restriction, and perceived disease burden, rather than localized neurobiological mechanisms. Accordingly, these findings should be interpreted as differential predictive contributions within a population-based model rather than evidence of region-specific causal pathways.

From a modeling perspective, Support Vector Machine and Gaussian Naïve Bayes achieved a relatively balanced performance in terms of discrimination and calibration. The integration of SHAP substantially enhanced model transparency by enabling visualization of feature-level contributions at both global and individual levels. The identification of lower limb pain and BMI as consistently influential predictors highlights the potential value of combining somatic symptom profiles with anthropometric indicators in future research on depression risk stratification and phenotypic characterization.

It should be noted, however, that despite acceptable overall discrimination and specificity, recall and F1-scores for depressive individuals remained relatively modest, indicating limited sensitivity of the current models for identifying depressive cases. As a result, the present findings are more suitable for methodological exploration and risk pattern characterization than for direct clinical screening or early detection. Future methodological work incorporating strategies to address outcome imbalance may further improve sensitivity.

Several limitations warrant consideration. First, the observational study design precludes conclusions regarding causal directionality between pain and depression, and reverse causation cannot be excluded. Second, pain measures in CHARLS were self-reported, which may introduce reporting bias and limit the assessment of pain severity or subtype. Third, the absence of biological markers, such as inflammatory or neuroendocrine indicators, constrains deeper interpretation of potential biological underpinnings.

Despite these limitations, the study provides population-level evidence supporting the importance of considering somatic symptom profiles alongside sociodemographic factors when examining depression-related risk in aging populations. Rather than serving as stand-alone clinical tools, interpretable machine learning models of this type may inform hypothesis generation, risk stratification research, and the design of integrated observational frameworks that bridge physical and mental health domains.

## Supporting information

S1 File CHARLS variables and coding schemes used in the analysis. This table summarizes all variables derived from the China Health and Retirement Longitudinal Study (CHARLS) that were included in the final analyses, including variable names, original CHARLS variable identifiers, definitions, and coding schemes.
(XLSX)

S2 File Illustrative analysis workflow. This file provides an illustrative and runnable Python analysis workflow in an accessible document format, including data preprocessing, feature selection, model training, hyperparameter tuning, performance evaluation, and SHAP-based interpretation.
(DOCX)
