# Supplementary material for: Exploration of comorbidity mechanisms between chronic pain and depression: Machine learning prediction models and SHAP interpretability analysis based on the CHARLS cohort
Source: PLoS One. 2026 Jun 8;21(6):e0349135. doi: 10.1371/journal.pone.0349135 (PMC13245799; doi:10.1371/journal.pone.0349135)
Supplement: S2 File — This file provides an illustrative and runnable Python analysis workflow in an accessible document format, including data preprocessing, feature selection, model training, hyperparameter tuning, performance evaluation, and SHAP-based interpretation. (DOCX) [file pone.0349135.s002.docx]

**File S2. Illustrative analysis workflow**

This supplementary file provides a readable Word version of the Python workflow. The code can be run after copying it into a .py file or by using the accompanying File_S2_runnable.py script. Example command: python File_S2_runnable.py --input analysis_data.csv --outdir outputs

0001 #!/usr/bin/env python3

0002 # -*- coding: utf-8 -*-

0003 """

0004 File S2. Illustrative analysis workflow

0005

0006 This script provides an illustrative and reproducible workflow for the analysis of

0007 chronic pain sites, mental health/depression status, machine-learning prediction,

0008 and SHAP-based model interpretation.

0009

0010 Recommended command:

0011 python File_S2_runnable.py --input analysis_data.csv --outdir outputs

0012

0013 Notes:

0014 1. The input CSV should contain the variables listed in EXPECTED_COLUMNS.

0015 2. If the original file uses the name "前期sharp分析.csv", run:

0016 python File_S2_runnable.py --input "前期sharp分析.csv" --outdir outputs

0017 3. XGBoost and SHAP analyses are skipped automatically if the corresponding

0018 packages are not installed.

0019 """

0020

0021 import argparse

0022 import json

0023 import os

0024 import warnings

0025 from pathlib import Path

0026

0027 import numpy as np

0028 import pandas as pd

0029

0030 import matplotlib

0031 matplotlib.use("Agg")

0032 import matplotlib.pyplot as plt

0033

0034 from sklearn.base import clone

0035 from sklearn.calibration import calibration_curve

0036 from sklearn.ensemble import RandomForestClassifier

0037 from sklearn.feature_selection import RFE

0038 from sklearn.linear_model import Lasso, LogisticRegression

0039 from sklearn.metrics import (

0040 accuracy_score,

0041 auc,

0042 classification_report,

0043 confusion_matrix,

0044 roc_auc_score,

0045 roc_curve,

0046 )

0047 from sklearn.model_selection import GridSearchCV, train_test_split

0048 from sklearn.naive_bayes import BernoulliNB, GaussianNB

0049 from sklearn.neighbors import KNeighborsClassifier

0050 from sklearn.pipeline import Pipeline

0051 from sklearn.preprocessing import LabelEncoder, StandardScaler

0052 from sklearn.svm import SVC

0053

0054 warnings.filterwarnings("ignore")

0055 plt.rcParams["axes.unicode_minus"] = False

0056

0057 EXPECTED_COLUMNS = [

0058 "AGE", "EDUCATION", "MARRIAGE", "PLACE OF RESIDENCE", "DRINKING", "SMOKING",

0059 "BMI", "HEADACHE", "SHOULDER PAIN", "ARM PAIN", "WRIST PAIN", "FINGER PAIN",

0060 "CHEST PAIN", "STOMACHACHE", "BACK PAIN", "LOW BACK PAIN", "HIP PAIN",

0061 "LEG PAIN", "KNEE PAIN", "ANKLE PAIN", "TOE PAIN", "NECK PAIN", "MENTAL HEALTH"

0062 ]

0063

0064 DEFAULT_FINAL_FEATURES = [

0065 "LEG PAIN", "HEADACHE", "SHOULDER PAIN", "ARM PAIN",

0066 "BMI", "KNEE PAIN", "EDUCATION", "LOW BACK PAIN"

0067 ]

0068

0069

0070 def parse_args():

0071 parser = argparse.ArgumentParser(description="Illustrative pain-depression machine-learning workflow")

0072 parser.add_argument("--input", required=True, help="Path to the input CSV file")

0073 parser.add_argument("--outdir", default="outputs", help="Directory for output tables and figures")

0074 parser.add_argument("--test-size", type=float, default=0.20, help="Test-set proportion")

0075 parser.add_argument("--random-state", type=int, default=42, help="Random seed")

0076 return parser.parse_args()

0077

0078

0079 def require_columns(data, columns):

0080 missing = [col for col in columns if col not in data.columns]

0081 if missing:

0082 raise ValueError(

0083 "The input file is missing required columns: " + ", ".join(missing)

0084 )

0085

0086

0087 def load_and_prepare_data(input_path):

0088 data = pd.read_csv(input_path)

0089 data = data.drop_duplicates(keep="first").copy()

0090

0091 # Harmonize sex/gender coding. The workflow accepts either GENDER or MALE/FEMALE.

0092 if "GENDER" in data.columns:

0093 data["GENDER"] = data["GENDER"].replace({"M": "Male", "F": "Female", 1: "Male", 2: "Female"})

0094 data = pd.get_dummies(data, columns=["GENDER"], dtype=int)

0095 data = data.rename(columns={"GENDER_Male": "MALE", "GENDER_Female": "FEMALE"})

0096 elif "MALE" not in data.columns or "FEMALE" not in data.columns:

0097 raise ValueError("The input file must contain either GENDER or both MALE and FEMALE columns.")

0098

0099 required = ["MALE", "FEMALE"] + EXPECTED_COLUMNS

0100 require_columns(data, required)

0101

0102 # Keep the analytical variables in a fixed order.

0103 ordered_columns = [

0104 "AGE", "MALE", "FEMALE", "EDUCATION", "MARRIAGE", "PLACE OF RESIDENCE",

0105 "DRINKING", "SMOKING", "BMI", "HEADACHE", "SHOULDER PAIN", "ARM PAIN",

0106 "WRIST PAIN", "FINGER PAIN", "CHEST PAIN", "STOMACHACHE", "BACK PAIN",

0107 "LOW BACK PAIN", "HIP PAIN", "LEG PAIN", "KNEE PAIN", "ANKLE PAIN",

0108 "TOE PAIN", "NECK PAIN", "MENTAL HEALTH"

0109 ]

0110 data = data[ordered_columns].copy()

0111

0112 # Convert the target into 0/1 numeric labels if necessary.

0113 if not pd.api.types.is_numeric_dtype(data["MENTAL HEALTH"]):

0114 label_encoder = LabelEncoder()

0115 data["MENTAL HEALTH"] = label_encoder.fit_transform(data["MENTAL HEALTH"].astype(str))

0116

0117 # Ensure numeric predictors and remove records with incomplete analytical variables.

0118 for col in ordered_columns:

0119 data[col] = pd.to_numeric(data[col], errors="coerce")

0120 data = data.dropna(axis=0, how="any").reset_index(drop=True)

0121

0122 unique_target = sorted(data["MENTAL HEALTH"].dropna().unique().tolist())

0123 if len(unique_target) != 2:

0124 raise ValueError("MENTAL HEALTH must be a binary outcome after preprocessing.")

0125

0126 return data

0127

0128

0129 def run_feature_selection(x, y, random_state):

0130 scaler = StandardScaler()

0131 x_scaled = scaler.fit_transform(x)

0132

0133 lasso = Lasso(random_state=random_state, max_iter=10000)

0134 parameters = {"alpha": np.logspace(-4, 0, 50)}

0135 lasso_regressor = GridSearchCV(

0136 lasso,

0137 parameters,

0138 scoring="neg_mean_squared_error",

0139 cv=5,

0140 n_jobs=1,

0141 )

0142 lasso_regressor.fit(x_scaled, y)

0143 best_lasso = lasso_regressor.best_estimator_

0144 lasso_importance = np.abs(best_lasso.coef_)

0145 lasso_features = list(x.columns[lasso_importance > 0])

0146

0147 rf = RandomForestClassifier(n_estimators=100, random_state=random_state, n_jobs=1)

0148 n_features_to_select = min(10, len(x.columns))

0149 rfe = RFE(estimator=rf, n_features_to_select=n_features_to_select, step=1)

0150 rfe.fit(x, y)

0151 rfe_features = list(x.columns[rfe.support_])

0152

0153 common_features = sorted(set(lasso_features).intersection(set(rfe_features)))

0154

0155 feature_importance = pd.DataFrame({

0156 "feature": x.columns,

0157 "lasso_importance": lasso_importance,

0158 "rfe_ranking": rfe.ranking_,

0159 })

0160 max_rank = feature_importance["rfe_ranking"].max()

0161 feature_importance["rfe_importance"] = (

0162 max_rank - feature_importance["rfe_ranking"] + 1

0163 ) / max_rank

0164 if feature_importance["lasso_importance"].max() > 0:

0165 feature_importance["lasso_importance"] = (

0166 feature_importance["lasso_importance"] / feature_importance["lasso_importance"].max()

0167 )

0168 feature_importance["selected_by_both"] = feature_importance["feature"].isin(common_features)

0169

0170 return {

0171 "lasso_features": lasso_features,

0172 "rfe_features": rfe_features,

0173 "common_features": common_features,

0174 "feature_importance": feature_importance,

0175 "best_lasso_alpha": float(lasso_regressor.best_params_["alpha"]),

0176 }

0177

0178

0179 def choose_final_features(available_features, common_features):

0180 prespecified = [feature for feature in DEFAULT_FINAL_FEATURES if feature in available_features]

0181 if prespecified:

0182 return prespecified

0183 if common_features:

0184 return common_features

0185 return list(available_features[: min(8, len(available_features))])

0186

0187

0188 def get_models(random_state):

0189 models = {

0190 "Logistic Regression": Pipeline([

0191 ("scaler", StandardScaler()),

0192 ("model", LogisticRegression(max_iter=2000, random_state=random_state)),

0193 ]),

0194 "Gaussian Naive Bayes": Pipeline([

0195 ("scaler", StandardScaler()),

0196 ("model", GaussianNB()),

0197 ]),

0198 "Bernoulli Naive Bayes": BernoulliNB(),

0199 "Support Vector Machine": Pipeline([

0200 ("scaler", StandardScaler()),

0201 ("model", SVC(C=100, gamma=0.002, probability=True, random_state=random_state)),

0202 ]),

0203 "Random Forest": RandomForestClassifier(n_estimators=100, random_state=random_state, n_jobs=1),

0204 "K-Nearest Neighbors": Pipeline([

0205 ("scaler", StandardScaler()),

0206 ("model", KNeighborsClassifier(n_neighbors=5)),

0207 ]),

0208 }

0209

0210 try:

0211 from xgboost import XGBClassifier

0212 models["XGBoost"] = XGBClassifier(

0213 n_estimators=100,

0214 learning_rate=0.05,

0215 max_depth=3,

0216 subsample=0.8,

0217 colsample_bytree=0.8,

0218 eval_metric="logloss",

0219 random_state=random_state,

0220 n_jobs=1,

0221 )

0222 except Exception:

0223 pass

0224

0225 return models

0226

0227

0228 def predicted_probabilities(model, x_test):

0229 if hasattr(model, "predict_proba"):

0230 return model.predict_proba(x_test)[:, 1]

0231 if hasattr(model, "decision_function"):

0232 scores = model.decision_function(x_test)

0233 return (scores - scores.min()) / (scores.max() - scores.min())

0234 raise ValueError("The model does not provide probabilities or decision scores.")

0235

0236

0237 def evaluate_models(x_train, x_test, y_train, y_test, random_state, outdir):

0238 models = get_models(random_state)

0239 records = []

0240 fitted_models = {}

0241 reports = {}

0242

0243 for name, model in models.items():

0244 fitted = clone(model)

0245 fitted.fit(x_train, y_train)

0246 y_pred = fitted.predict(x_test)

0247 y_prob = predicted_probabilities(fitted, x_test)

0248

0249 records.append({

0250 "model": name,

0251 "accuracy": accuracy_score(y_test, y_pred),

0252 "auc": roc_auc_score(y_test, y_prob),

0253 })

0254 fitted_models[name] = fitted

0255 reports[name] = {

0256 "confusion_matrix": confusion_matrix(y_test, y_pred).tolist(),

0257 "classification_report": classification_report(y_test, y_pred, output_dict=True),

0258 }

0259

0260 performance = pd.DataFrame(records).sort_values("auc", ascending=False)

0261 performance.to_csv(outdir / "model_performance.csv", index=False)

0262 with open(outdir / "classification_reports.json", "w", encoding="utf-8") as f:

0263 json.dump(reports, f, indent=2, ensure_ascii=False)

0264

0265 return fitted_models, performance

0266

0267

0268 def plot_feature_selection(feature_importance, outdir):

0269 plot_data = feature_importance.sort_values(

0270 ["selected_by_both", "lasso_importance"], ascending=[False, False]

0271 )

0272 y_pos = np.arange(len(plot_data))

0273 height = 0.35

0274

0275 fig, ax = plt.subplots(figsize=(10, max(6, len(plot_data) * 0.35)))

0276 ax.barh(y_pos - height / 2, plot_data["lasso_importance"], height, label="LASSO importance")

0277 ax.barh(y_pos + height / 2, plot_data["rfe_importance"], height, label="RFE importance")

0278 ax.set_yticks(y_pos)

0279 ax.set_yticklabels(plot_data["feature"])

0280 ax.invert_yaxis()

0281 ax.set_xlabel("Normalized importance")

0282 ax.set_title("Feature importance from LASSO and RFE")

0283 ax.legend()

0284 fig.tight_layout()

0285 fig.savefig(outdir / "feature_importance_comparison.png", dpi=300, bbox_inches="tight")

0286 plt.close(fig)

0287

0288

0289 def plot_model_performance(performance, outdir):

0290 fig, ax = plt.subplots(figsize=(10, 6))

0291 x_pos = np.arange(len(performance))

0292 ax.bar(x_pos, performance["auc"])

0293 ax.set_xticks(x_pos)

0294 ax.set_xticklabels(performance["model"], rotation=35, ha="right")

0295 ax.set_ylabel("AUC")

0296 ax.set_ylim(0, 1)

0297 ax.set_title("Model discrimination performance")

0298 for idx, value in enumerate(performance["auc"]):

0299 ax.text(idx, value + 0.01, f"{value:.3f}", ha="center", fontsize=9)

0300 fig.tight_layout()

0301 fig.savefig(outdir / "model_auc_comparison.png", dpi=300, bbox_inches="tight")

0302 plt.close(fig)

0303

0304

0305 def plot_roc_curves(models, x_test, y_test, outdir):

0306 fig, ax = plt.subplots(figsize=(8, 7))

0307 for name, model in models.items():

0308 y_prob = predicted_probabilities(model, x_test)

0309 fpr, tpr, _ = roc_curve(y_test, y_prob)

0310 roc_auc = auc(fpr, tpr)

0311 ax.plot(fpr, tpr, lw=2, label=f"{name} (AUC = {roc_auc:.3f})")

0312 ax.plot([0, 1], [0, 1], linestyle="--", lw=1)

0313 ax.set_xlabel("False positive rate")

0314 ax.set_ylabel("True positive rate")

0315 ax.set_title("Receiver operating characteristic curves")

0316 ax.legend(loc="lower right", fontsize=8)

0317 fig.tight_layout()

0318 fig.savefig(outdir / "roc_curves.png", dpi=300, bbox_inches="tight")

0319 plt.close(fig)

0320

0321

0322 def plot_calibration_curves(models, x_test, y_test, outdir):

0323 fig, ax = plt.subplots(figsize=(8, 7))

0324 ax.plot([0, 1], [0, 1], linestyle="--", label="Perfect calibration")

0325 for name, model in models.items():

0326 y_prob = predicted_probabilities(model, x_test)

0327 fraction_of_positives, mean_predicted_value = calibration_curve(y_test, y_prob, n_bins=10)

0328 ax.plot(mean_predicted_value, fraction_of_positives, marker="o", label=name)

0329 ax.set_xlabel("Mean predicted probability")

0330 ax.set_ylabel("Fraction of positives")

0331 ax.set_title("Calibration curves")

0332 ax.legend(loc="best", fontsize=8)

0333 fig.tight_layout()

0334 fig.savefig(outdir / "calibration_curves.png", dpi=300, bbox_inches="tight")

0335 plt.close(fig)

0336

0337

0338 def calculate_net_benefit(threshold, y_true, y_pred_prob):

0339 y_pred = (y_pred_prob >= threshold).astype(int)

0340 true_positive = np.sum((y_pred == 1) & (y_true == 1))

0341 false_positive = np.sum((y_pred == 1) & (y_true == 0))

0342 n = len(y_true)

0343 return (true_positive / n) - (false_positive / n) * (threshold / (1 - threshold))

0344

0345

0346 def plot_decision_curves(models, x_test, y_test, outdir):

0347 thresholds = np.arange(0.01, 0.99, 0.01)

0348 y_array = np.asarray(y_test)

0349

0350 fig, ax = plt.subplots(figsize=(8, 7))

0351 treat_all = [np.mean(y_array) - (1 - np.mean(y_array)) * (t / (1 - t)) for t in thresholds]

0352 treat_none = np.zeros_like(thresholds)

0353 ax.plot(thresholds, treat_all, linestyle="--", label="Treat all")

0354 ax.plot(thresholds, treat_none, linestyle="-", label="Treat none")

0355

0356 for name, model in models.items():

0357 y_prob = predicted_probabilities(model, x_test)

0358 net_benefit = [calculate_net_benefit(t, y_array, y_prob) for t in thresholds]

0359 ax.plot(thresholds, net_benefit, label=name)

0360

0361 ax.set_xlabel("Threshold probability")

0362 ax.set_ylabel("Net benefit")

0363 ax.set_title("Decision curve analysis")

0364 ax.legend(loc="best", fontsize=8)

0365 fig.tight_layout()

0366 fig.savefig(outdir / "decision_curve_analysis.png", dpi=300, bbox_inches="tight")

0367 plt.close(fig)

0368

0369

0370 def plot_shap_if_available(models, x_test, outdir):

0371 if "XGBoost" not in models:

0372 return "SHAP analysis skipped because XGBoost was not available."

0373

0374 try:

0375 import shap

0376 except Exception:

0377 return "SHAP analysis skipped because the shap package was not available."

0378

0379 model = models["XGBoost"]

0380 explainer = shap.Explainer(model)

0381 shap_values = explainer(x_test)

0382

0383 shap.summary_plot(shap_values, x_test, plot_type="bar", show=False)

0384 plt.title("SHAP feature importance")

0385 plt.tight_layout()

0386 plt.savefig(outdir / "shap_feature_importance.png", dpi=300, bbox_inches="tight")

0387 plt.close()

0388

0389 shap.summary_plot(shap_values, x_test, show=False)

0390 plt.title("SHAP summary plot")

0391 plt.tight_layout()

0392 plt.savefig(outdir / "shap_summary_plot.png", dpi=300, bbox_inches="tight")

0393 plt.close()

0394

0395 important_features = [feature for feature in DEFAULT_FINAL_FEATURES if feature in x_test.columns][:3]

0396 for feature in important_features:

0397 shap.dependence_plot(feature, shap_values.values, x_test, show=False)

0398 plt.tight_layout()

0399 safe_name = feature.replace(" ", "_").replace("/", "_")

0400 plt.savefig(outdir / f"shap_dependence_{safe_name}.png", dpi=300, bbox_inches="tight")

0401 plt.close()

0402

0403 return "SHAP analysis completed."

0404

0405

0406 def main():

0407 args = parse_args()

0408 outdir = Path(args.outdir)

0409 outdir.mkdir(parents=True, exist_ok=True)

0410

0411 data = load_and_prepare_data(args.input)

0412 x_all = data.drop(columns=["MENTAL HEALTH"])

0413 y = data["MENTAL HEALTH"].astype(int)

0414

0415 feature_selection = run_feature_selection(x_all, y, args.random_state)

0416 feature_selection["feature_importance"].to_csv(outdir / "feature_selection_importance.csv", index=False)

0417 with open(outdir / "selected_features.json", "w", encoding="utf-8") as f:

0418 json.dump({

0419 "best_lasso_alpha": feature_selection["best_lasso_alpha"],

0420 "lasso_features": feature_selection["lasso_features"],

0421 "rfe_features": feature_selection["rfe_features"],

0422 "common_features": feature_selection["common_features"],

0423 }, f, indent=2, ensure_ascii=False)

0424 plot_feature_selection(feature_selection["feature_importance"], outdir)

0425

0426 final_features = choose_final_features(x_all.columns, feature_selection["common_features"])

0427 x = data[final_features].copy()

0428 x_train, x_test, y_train, y_test = train_test_split(

0429 x,

0430 y,

0431 test_size=args.test_size,

0432 random_state=args.random_state,

0433 stratify=y,

0434 )

0435

0436 fitted_models, performance = evaluate_models(

0437 x_train, x_test, y_train, y_test, args.random_state, outdir

0438 )

0439 plot_model_performance(performance, outdir)

0440 plot_roc_curves(fitted_models, x_test, y_test, outdir)

0441 plot_calibration_curves(fitted_models, x_test, y_test, outdir)

0442 plot_decision_curves(fitted_models, x_test, y_test, outdir)

0443 shap_status = plot_shap_if_available(fitted_models, x_test, outdir)

0444

0445 summary = {

0446 "n_samples": int(len(data)),

0447 "n_features_all": int(x_all.shape[1]),

0448 "final_features": final_features,

0449 "best_model_by_auc": performance.iloc[0].to_dict(),

0450 "shap_status": shap_status,

0451 }

0452 with open(outdir / "analysis_summary.json", "w", encoding="utf-8") as f:

0453 json.dump(summary, f, indent=2, ensure_ascii=False)

0454

0455 print("Analysis completed successfully.")

0456 print(json.dumps(summary, indent=2, ensure_ascii=False))

0457

0458

0459 if __name__ == "__main__":

0460 main()
