# Supplementary material for: Exploration of comorbidity mechanisms between chronic pain and depression: Machine learning prediction models and SHAP interpretability analysis based on the CHARLS cohort
Source: PLoS One. 2026 Jun 8;21(6):e0349135. doi: 10.1371/journal.pone.0349135 (PMC13245799; doi:10.1371/journal.pone.0349135)
Supplement: Attachment — Submitted filename: Response to Reviewers.docx [file pone.0349135.s004.docx]

Dear Dr. Pedrammehr,

Thank you for the opportunity to revise our manuscript titled “Exploration of comorbidity mechanisms between chronic pain and depression: Machine learning prediction models and SHAP interpretability analysis based on the CHARLS cohort” (ID:PONE-D-25-30274). We appreciate the time and effort that you and the reviewers have dedicated to providing valuable feedback. The comments have been instrumental in improving the quality of our paper. We have carefully considered all the suggestions and made revisions accordingly. Below, we provide a point-by-point response to each of the reviewers’ comments.

Response to Editorial Requirements

1. PLOS ONE style and file naming requirements

Response:

Thank you for the reminder. We have carefully revised the manuscript to ensure full compliance with PLOS ONE’s style requirements, including formatting, structure, and file naming conventions. The main text, title page, and author affiliations have been checked against the official PLOS ONE templates, and all files have been renamed according to the journal’s guidelines. We believe that the revised submission now fully adheres to PLOS ONE formatting standards.

2. Data repository and Data Availability Statement

Response:

Thank you for pointing this out. We acknowledge that the repository originally indicated in the Data Availability Statement did not meet PLOS ONE’s repository requirements.

In response, we have deposited the minimal dataset necessary to reproduce the main findings of this study in a stable, public repository (figshare). The dataset is publicly accessible and has been assigned a permanent DOI. We have updated the Data Availability Statement in the manuscript to include the corresponding URL and DOI (Data Availability Statement, page 19, lines 389–390).

3. Supporting Information captions and in-text citations

Response:

We appreciate this clarification. We have now added complete captions for all Supporting Information files at the end of the manuscript, in accordance with PLOS ONE guidelines. In addition, all in-text citations referring to the Supporting Information have been carefully checked and updated to ensure consistency with the revised captions.

Response to Reviewer #1

1.	Comment 1:First, the performance imbalance between depressive and non-depressive classification requires urgent attention. While model accuracy and specificity are reasonable, recall and F1 scores for depressive individuals are consistently low, indicating poor sensitivity. This weakens the applicability of the models for screening or early detection purposes. The authors should consider applying techniques such as class weighting, oversampling (e.g., SMOTE), or adjusting the decision threshold to address class imbalance. If these are not feasible within the current analysis, the authors must clearly acknowledge this limitation in both the abstract and discussion and refrain from suggesting clinical implementation without qualification.

Response: Thank you for this important and constructive comment. We agree that the imbalance between depressive and non-depressive classes resulted in limited sensitivity, as reflected by relatively low recall and F1-scores for depressive individuals, which constrains the applicability of the models for screening or early detection.

In the current analysis, we did not further implement class weighting, oversampling techniques (e.g., SMOTE), or decision threshold adjustment, as the primary aim of this study was to explore depression-related risk patterns using interpretable machine learning models rather than to optimize screening performance. In accordance with the reviewer’s suggestion, we have explicitly acknowledged this limitation in both the Abstract and the Discussion. Specifically, we now state that the models demonstrate limited sensitivity for depressive cases and are therefore more suitable for population-level risk pattern characterization and hypothesis generation, rather than direct clinical screening or early detection (Abstract, page 2, lines 37–40; Discussion, page 18, lines 345–350).

In addition, all statements implying unqualified clinical implementation have been revised or removed to avoid overinterpretation. We believe these revisions appropriately address the reviewer’s concern while clarifying the scope and limitations of the present study.

2. Comment 2:Second, the interpretation of SHAP results should be refined and made more consistent. In particular, the discussion around education level as a protective factor contradicts the assertion in the SHAP analysis that its contribution is minimal. The authors need to either reconcile these findings with a clearer explanation or revise the interpretation to match the quantitative results. Additionally, the SHAP plots should be accompanied by more intuitive summaries, possibly including numerical summaries or stratified effects across risk groups.

Response: Thank you for this insightful comment. We agree that the initial interpretation of the SHAP results, particularly regarding education level, required refinement to ensure consistency with the quantitative findings.

In response, we have revised the SHAP-related interpretation throughout the manuscript to align more closely with the magnitude of the SHAP contributions. Specifically, while higher education level showed a directionally protective association, we now clarify that its relative predictive contribution was modest compared with pain-related features. Accordingly, education is no longer described as a dominant protective factor, but rather as a secondary contributor within the multivariable prediction framework (Discussion, page 16, paragraph lines 316–322).

In addition, to enhance interpretability beyond the SHAP plots, we have provided more intuitive summaries of the SHAP results. We added a narrative and numerical summary of feature importance and conducted a risk-stratified SHAP analysis, comparing key predictors across low-, moderate-, and high-risk groups. This stratified presentation highlights the heterogeneity of feature contributions across risk levels and facilitates a clearer understanding of the relative importance of pain-related symptoms, BMI, and education (Results, page 15, lines287–301).

We believe these revisions resolve the inconsistency noted by the reviewer and substantially improve the clarity and interpretability of the SHAP-based findings.

3. Comment 3:Third, the language of the manuscript requires modest revision to improve clarity and accuracy. While generally intelligible, the manuscript contains several lengthy and complex sentences that obscure meaning. These should be rewritten for conciseness and clarity. Furthermore, the authors should eliminate or rephrase any causal language, such as "mechanism," "regulatory role," or "explains the pathway," as the study is observational and not designed to infer causation. Appropriate phrasing should refer to "associations" or "predictive contributions."

Response:Thank you for this helpful comment. We agree that further refinement of the language was necessary to improve clarity, precision, and consistency with the observational nature of the study.

In response, we conducted a thorough language revision of the manuscript. Specifically, lengthy and complex sentences were rewritten to improve conciseness and readability, and ambiguous or potentially confusing phrasing was clarified throughout the text. In addition, all causal or mechanistic language—including terms such as “mechanism,” “regulatory role,” and “explains the pathway”—has been removed or rephrased. These expressions were replaced with terminology more appropriate for an observational and predictive study design, such as “association,” “predictive contribution,” or “risk pattern.”

These revisions were applied consistently across the Abstract, Results, and Discussion sections . We believe that these changes substantially improve the clarity of the manuscript and ensure that the interpretation of findings remains appropriately cautious and methodologically accurate.

4. Comment 4:Fourth, the description of the statistical pipeline could benefit from greater transparency. While the authors note the use of Bayesian optimization for hyperparameter tuning and stratified train/test splits, details on how the split was stratified (e.g., based on class distribution) and whether cross-validation was used for model robustness should be explicitly stated. Including a supplemental methods appendix with the full model training pipeline and Python/R code would enhance reproducibility.

Response:Thank you for this constructive comment. We agree that greater clarity regarding the statistical pipeline would improve transparency and reproducibility.

In response, we have substantially expanded the description of the analytical workflow in a newly added **Supplementary Methods** section. Specifically, we now explicitly state that the train/test split was performed using stratified sampling based on depression status to preserve class distribution, and that stratified cross-validation was applied during model training and hyperparameter tuning to enhance robustness under class imbalance. The use of Bayesian optimization, including its role in selecting optimal hyperparameters based on cross-validated performance, is also described in greater detail.

In addition, to further support transparency, we have included **Supplementary Code S1**, which provides an illustrative overview of the main analytical workflow (data preprocessing, feature selection, model training, hyperparameter tuning, and performance evaluation). This code is intended for illustrative purposes and does not represent a fully executable script, as some dataset-specific and environment-dependent components are omitted.

We believe that the combination of a detailed Supplementary Methods description and illustrative code sufficiently clarifies the statistical pipeline while remaining consistent with data governance requirements and the scope of the present study.

5. Comment 5:Fifth, while the data availability complies with PLOS ONE policies, it is recommended that the authors specify which CHARLS variables were used and provide their coding or labels in a supplementary table. This will support transparency for readers and future researchers aiming to replicate or extend the study.

Response:

Thank you for this helpful suggestion. We agree that explicitly documenting the variables used and their coding would further enhance transparency and facilitate reproducibility.

In response, we have added a new supplementary table summarizing all CHARLS variables included in the analysis, along with their corresponding labels, coding schemes, and brief descriptions. This table specifies how each variable was operationalized in the modeling process, including outcome definition, sociodemographic characteristics, pain-related variables.

The newly added table is provided as Supplementary Table 1, and it is referenced in the revised Methods section (Methods, page 6, lines124–125). We believe that this addition will assist readers and future researchers in replicating or extending the present analysis using CHARLS data, while remaining consistent with PLOS ONE data availability policies.

Response to Reviewer #2

1. Comment 1: .Please elaborate on the hyperparameter tuning process for each model. It is currently too vague.

Response:Thank you for this helpful comment. We agree that the description of the hyperparameter tuning process required further clarification.

In response, we have substantially expanded the description of hyperparameter optimization in the Methods section and Supplementary Methods. Specifically, hyperparameter tuning for all models was conducted using Bayesian optimization within the training dataset. For each model, a predefined search space was specified based on commonly recommended parameter ranges in the literature (e.g., regularization strength for logistic regression, kernel parameters for SVM, number of estimators and tree depth for ensemble models, and neighborhood size for KNN). Model performance during tuning was evaluated using stratified cross-validation to ensure robustness under class imbalance.

The optimal hyperparameter configurations were selected based on average cross-validation performance, rather than single-split results, to reduce the risk of overfitting. A concise summary of the tuning strategy and key hyperparameters for each model has now been explicitly described in the revised manuscript (Methods, page 6-7, lines 144–165; Supplementary Methods, page 22).

We believe that these revisions provide sufficient transparency regarding the hyperparameter tuning process while maintaining clarity and reproducibility of the analytical workflow.

2.	Comment 2: Include ROC curves or AUC scores in addition to the reported metrics. This is a common practice in classification tasks.

Response: Thank you for this helpful suggestion. We agree that including ROC curves and AUC values provides a more comprehensive and threshold-independent evaluation of model performance.

In response, we have added ROC curves for all machine learning models and reported the corresponding AUC values in the revised manuscript. These metrics complement accuracy, precision, recall, and F1-score, and allow for a clearer comparison of the models’ discriminative ability, particularly under class imbalance. The ROC curves are presented in Figure 4, and the AUC values are summarized in Table 2 and described in the Results section (Results, page 12-13, lines 211–240).

We believe that the inclusion of ROC curves and AUC scores aligns the evaluation with common practice in classification studies and improves the interpretability and robustness of the model performance assessment.

3.Comment 3：The manuscript would benefit from restructuring the Discussion to more clearly separate interpretation from policy recommendations.

Response: Thank you for this valuable suggestion. We agree that a clearer separation between interpretation of findings and broader implications would improve the structure and readability of the Discussion.

In response, we have reorganized the Discussion section to more clearly distinguish between interpretation of the empirical results and their potential public health or policy implications. Specifically, we revised the early part of the Discussion to focus on interpreting the main findings in relation to existing literature and the predictive modeling framework, while methodological considerations and limitations are discussed separately. Broader implications are now presented in a distinct subsection and framed cautiously as potential implications rather than direct recommendations.

These revisions help ensure that interpretation remains grounded in the study’s observational and predictive nature, while policy-related considerations are clearly delineated and appropriately qualified (Discussion, page 16-18).

We believe that this restructuring improves conceptual clarity and aligns the Discussion more closely with the reviewer’s recommendation.

4. Comment 4：Add the sample size (n=38,970) to the Abstract for context.

Response: Thank you for this suggestion. We agree that explicitly reporting the sample size improves clarity and provides important context for the study.

Accordingly, we have added the sample size (n = 38,970) to the Abstract in the Methods section, where the study population is described (Abstract, page 1X, lines 22). We believe this revision enhances the transparency and readability of the Abstract.

5. Comment 5：The term “biphasic regulation” used for BMI should be briefly explained in lay terms.

Response:Thank you for this helpful suggestion. We agree that the term “biphasic regulation” may not be immediately clear to all readers and would benefit from a brief explanation in more accessible language.

In response, we have added a short clarification in the manuscript to explain that “biphasic regulation” refers to a non-linear, U-shaped association, whereby both relatively low and relatively high BMI values are associated with higher predicted depression risk, while intermediate BMI levels are associated with lower risk (Results, page 14, lines 259–265). This explanation is intended to improve readability and ensure that the concept is easily understood by a broad audience.

6. Comment 6：There are slso some typos and grammatical errors within the text.

Response:Response:Thank you for pointing this out. We have carefully proofread the entire manuscript and corrected typographical errors, grammatical mistakes, and minor language inconsistencies throughout the text. In addition, several sentences were revised to improve clarity and readability. We believe that these revisions have substantially improved the overall language quality of the manuscript.

We hope that these revisions meet your expectations and improve our manuscript significantly. Thank you once again for your constructive feedback.

We look forward to your response.

Best regards,

Jun-Jun Liu

Affiliated Hospital 6 of Nantong University

ycsdsrmyyybc@163.com
