# Supplementary material for: Exploration of comorbidity mechanisms between chronic pain and depression: Machine learning prediction models and SHAP interpretability analysis based on the CHARLS cohort
Source: PLoS One. 2026 Jun 8;21(6):e0349135. doi: 10.1371/journal.pone.0349135 (PMC13245799; doi:10.1371/journal.pone.0349135)
Supplement: Peer review history

# Author comment: Author response to Decision Letter 0

**DOI:** 10.1371/journal.pone.0349135.r001

16 Jun 2025

Attachment Submitted filename: PLOSOne_Human_Subjects_Research_Checklist.docx

# Peer review: Decision Letter 0

**DOI:** 10.1371/journal.pone.0349135.r002

**Authors:** Siamak Pedrammehr

11 Aug 2025

PONE-D-25-30274

Exploration of comorbidity mechanisms between chronic pain and depression: Machine learning prediction models and SHAP interpretability analysis based on the CHARLS cohort

PLOS ONE

Dear Dr. Liu,

Thank you for submitting your manuscript to PLOS ONE. After careful consideration, we feel that it has merit but does not fully meet PLOS ONE’s publication criteria as it currently stands. Therefore, we invite you to submit a revised version of the manuscript that addresses the points raised during the review process.

Please submit your revised manuscript by Sep 25 2025 11:59PM. If you will need more time than this to complete your revisions, please reply to this message or contact the journal office at plosone@plos.org. When you're ready to submit your revision, log on to https://www.editorialmanager.com/pone/ and select the 'Submissions Needing Revision' folder to locate your manuscript file.

Please include the following items when submitting your revised manuscript:

- A rebuttal letter that responds to each point raised by the academic editor and reviewer(s). You should upload this letter as a separate file labeled 'Response to Reviewers'.
- A marked-up copy of your manuscript that highlights changes made to the original version. You should upload this as a separate file labeled 'Revised Manuscript with Track Changes'.
- An unmarked version of your revised paper without tracked changes. You should upload this as a separate file labeled 'Manuscript'.

If you would like to make changes to your financial disclosure, please include your updated statement in your cover letter. Guidelines for resubmitting your figure files are available below the reviewer comments at the end of this letter.

If applicable, we recommend that you deposit your laboratory protocols in protocols.io to enhance the reproducibility of your results. Protocols.io assigns your protocol its own identifier (DOI) so that it can be cited independently in the future. For instructions see: https://journals.plos.org/plosone/s/submission-guidelines#loc-laboratory-protocols. Additionally, PLOS ONE offers an option for publishing peer-reviewed Lab Protocol articles, which describe protocols hosted on protocols.io. Read more information on sharing protocols at https://plos.org/protocols?utm_medium=editorial-email&utm_source=authorletters&utm_campaign=protocols.

We look forward to receiving your revised manuscript.

Kind regards,

Siamak Pedrammehr, Ph.D.

Academic Editor

PLOS ONE

Journal Requirements:

When submitting your revision, we need you to address these additional requirements.

1. Please ensure that your manuscript meets PLOS ONE's style requirements, including those for file naming. The PLOS ONE style templates can be found at

https://journals.plos.org/plosone/s/file?id=wjVg/PLOSOne_formatting_sample_main_body.pdf and

https://journals.plos.org/plosone/s/file?id=ba62/PLOSOne_formatting_sample_title_authors_affiliations.pdf

2. Thank you for uploading your study's underlying data set. Unfortunately, the repository you have noted in your Data Availability statement does not qualify as an acceptable data repository according to PLOS's standards.

At this time, please upload the minimal data set necessary to replicate your study's findings to a stable, public repository (such as figshare or Dryad) and provide us with the relevant URLs, DOIs, or accession numbers that may be used to access these data. For a list of recommended repositories and additional information on PLOS standards for data deposition, please see https://journals.plos.org/plosone/s/recommended-repositories.

3. Please include captions for your Supporting Information files at the end of your manuscript, and update any in-text citations to match accordingly. Please see our Supporting Information guidelines for more information: http://journals.plos.org/plosone/s/supporting-information.

4. If the reviewer comments include a recommendation to cite specific previously published works, please review and evaluate these publications to determine whether they are relevant and should be cited. There is no requirement to cite these works unless the editor has indicated otherwise.

[Note: HTML markup is below. Please do not edit.]

Reviewers' comments:

Reviewer's Responses to Questions

**Comments to the Author**

1. Is the manuscript technically sound, and do the data support the conclusions?

The manuscript must describe a technically sound piece of scientific research with data that supports the conclusions. Experiments must have been conducted rigorously, with appropriate controls, replication, and sample sizes. The conclusions must be drawn appropriately based on the data presented.

Reviewer #1: Yes

Reviewer #2: Yes

**********

2. Has the statistical analysis been performed appropriately and rigorously?

Reviewer #1: Yes

Reviewer #2: Yes

**********

3. Have the authors made all data underlying the findings in their manuscript fully available?

The PLOS Data policy requires authors to make all data underlying the findings described in their manuscript fully available without restriction, with rare exception (please refer to the Data Availability Statement in the manuscript PDF file). The data should be provided as part of the manuscript or its supporting information, or deposited to a public repository. For example, in addition to summary statistics, the data points behind means, medians and variance measures should be available. If there are restrictions on publicly sharing data—e.g. participant privacy or use of data from a third party—those must be specified.

Reviewer #1: No

Reviewer #2: Yes

**********

4. Is the manuscript presented in an intelligible fashion and written in standard English?

PLOS ONE does not copyedit accepted manuscripts, so the language in submitted articles must be clear, correct, and unambiguous. Any typographical or grammatical errors should be corrected at revision, so please note any specific errors here.

Reviewer #1: Yes

Reviewer #2: Yes

**********

5. Review Comments to the Author

Please use the space provided to explain your answers to the questions above. You may also include additional comments for the author, including concerns about dual publication, research ethics, or publication ethics. (Please upload your review as an attachment if it exceeds 20,000 characters)

Reviewer #1: First, the performance imbalance between depressive and non-depressive classification requires urgent attention. While model accuracy and specificity are reasonable, recall and F1 scores for depressive individuals are consistently low, indicating poor sensitivity. This weakens the applicability of the models for screening or early detection purposes. The authors should consider applying techniques such as class weighting, oversampling (e.g., SMOTE), or adjusting the decision threshold to address class imbalance. If these are not feasible within the current analysis, the authors must clearly acknowledge this limitation in both the abstract and discussion and refrain from suggesting clinical implementation without qualification.

Second, the interpretation of SHAP results should be refined and made more consistent. In particular, the discussion around education level as a protective factor contradicts the assertion in the SHAP analysis that its contribution is minimal. The authors need to either reconcile these findings with a clearer explanation or revise the interpretation to match the quantitative results. Additionally, the SHAP plots should be accompanied by more intuitive summaries, possibly including numerical summaries or stratified effects across risk groups.

Third, the language of the manuscript requires modest revision to improve clarity and accuracy. While generally intelligible, the manuscript contains several lengthy and complex sentences that obscure meaning. These should be rewritten for conciseness and clarity. Furthermore, the authors should eliminate or rephrase any causal language, such as "mechanism," "regulatory role," or "explains the pathway," as the study is observational and not designed to infer causation. Appropriate phrasing should refer to "associations" or "predictive contributions."

Fourth, the description of the statistical pipeline could benefit from greater transparency. While the authors note the use of Bayesian optimization for hyperparameter tuning and stratified train/test splits, details on how the split was stratified (e.g., based on class distribution) and whether cross-validation was used for model robustness should be explicitly stated. Including a supplemental methods appendix with the full model training pipeline and Python/R code would enhance reproducibility.

Fifth, while the data availability complies with PLOS ONE policies, it is recommended that the authors specify which CHARLS variables were used and provide their coding or labels in a supplementary table. This will support transparency for readers and future researchers aiming to replicate or extend the study.

Reviewer #2: this study examines the comorbidity mechanisms of chronic pain and depression in a large Chinese cohort using interpretable machine learning.However, several methodological and clarity issues need to be addressed before the manuscript can be considered for publication.

Major Points for Revision:

1.Please elaborate on the hyperparameter tuning process for each model. It is currently too vague.

2.Include ROC curves or AUC scores in addition to the reported metrics. This is a common practice in classification tasks.

3.The manuscript would benefit from restructuring the Discussion to more clearly separate interpretation from policy recommendations.

Minor Suggestions:

1.Add the sample size (n=38,970) to the Abstract for context.

2.The term “biphasic regulation” used for BMI should be briefly explained in lay terms.

There are slso some typos and grammatical errors within the text.

Ex:

1.“Headache demonstrated a significant left-skewed contribution distribution…”

**********

6. PLOS authors have the option to publish the peer review history of their article (what does this mean?). If published, this will include your full peer review and any attached files.

If you choose “no”, your identity will remain anonymous but your review may still be made public.

**Do you want your identity to be public for this peer review?** For information about this choice, including consent withdrawal, please see our Privacy Policy.

Reviewer #1: No

Reviewer #2: No

**********

[NOTE: If reviewer comments were submitted as an attachment file, they will be attached to this email and accessible via the submission site. Please log into your account, locate the manuscript record, and check for the action link "View Attachments". If this link does not appear, there are no attachment files.]

While revising your submission, please upload your figure files to the Preflight Analysis and Conversion Engine (PACE) digital diagnostic tool, https://pacev2.apexcovantage.com/. PACE helps ensure that figures meet PLOS requirements. To use PACE, you must first register as a user. Registration is free. Then, login and navigate to the UPLOAD tab, where you will find detailed instructions on how to use the tool. If you encounter any issues or have any questions when using PACE, please email PLOS at figures@plos.org. Please note that Supporting Information files do not need this step.

# Author comment: Author response to Decision Letter 1

**DOI:** 10.1371/journal.pone.0349135.r003

24 Feb 2026

Dear Dr. Pedrammehr,

Thank you for the opportunity to revise our manuscript titled “Exploration of comorbidity mechanisms between chronic pain and depression: Machine learning prediction models and SHAP interpretability analysis based on the CHARLS cohort” (ID:PONE-D-25-30274). We appreciate the time and effort that you and the reviewers have dedicated to providing valuable feedback. The comments have been instrumental in improving the quality of our paper. We have carefully considered all the suggestions and made revisions accordingly. Below, we provide a point-by-point response to each of the reviewers’ comments.

Response to Editorial Requirements

1. PLOS ONE style and file naming requirements

Response:

Thank you for the reminder. We have carefully revised the manuscript to ensure full compliance with PLOS ONE’s style requirements, including formatting, structure, and file naming conventions. The main text, title page, and author affiliations have been checked against the official PLOS ONE templates, and all files have been renamed according to the journal’s guidelines. We believe that the revised submission now fully adheres to PLOS ONE formatting standards.

2. Data repository and Data Availability Statement

Response:

Thank you for pointing this out. We acknowledge that the repository originally indicated in the Data Availability Statement did not meet PLOS ONE’s repository requirements.

In response, we have deposited the minimal dataset necessary to reproduce the main findings of this study in a stable, public repository (figshare). The dataset is publicly accessible and has been assigned a permanent DOI. We have updated the Data Availability Statement in the manuscript to include the corresponding URL and DOI (Data Availability Statement, page 19, lines 389–390).

3. Supporting Information captions and in-text citations

Response:

We appreciate this clarification. We have now added complete captions for all Supporting Information files at the end of the manuscript, in accordance with PLOS ONE guidelines. In addition, all in-text citations referring to the Supporting Information have been carefully checked and updated to ensure consistency with the revised captions.

Response to Reviewer #1

1. Comment 1:First, the performance imbalance between depressive and non-depressive classification requires urgent attention. While model accuracy and specificity are reasonable, recall and F1 scores for depressive individuals are consistently low, indicating poor sensitivity. This weakens the applicability of the models for screening or early detection purposes. The authors should consider applying techniques such as class weighting, oversampling (e.g., SMOTE), or adjusting the decision threshold to address class imbalance. If these are not feasible within the current analysis, the authors must clearly acknowledge this limitation in both the abstract and discussion and refrain from suggesting clinical implementation without qualification.

Response: Thank you for this important and constructive comment. We agree that the imbalance between depressive and non-depressive classes resulted in limited sensitivity, as reflected by relatively low recall and F1-scores for depressive individuals, which constrains the applicability of the models for screening or early detection.

In the current analysis, we did not further implement class weighting, oversampling techniques (e.g., SMOTE), or decision threshold adjustment, as the primary aim of this study was to explore depression-related risk patterns using interpretable machine learning models rather than to optimize screening performance. In accordance with the reviewer’s suggestion, we have explicitly acknowledged this limitation in both the Abstract and the Discussion. Specifically, we now state that the models demonstrate limited sensitivity for depressive cases and are therefore more suitable for population-level risk pattern characterization and hypothesis generation, rather than direct clinical screening or early detection (Abstract, page 2, lines 37–40; Discussion, page 18, lines 345–350).

In addition, all statements implying unqualified clinical implementation have been revised or removed to avoid overinterpretation. We believe these revisions appropriately address the reviewer’s concern while clarifying the scope and limitations of the present study.

2.Comment 2:Second, the interpretation of SHAP results should be refined and made more consistent. In particular, the discussion around education level as a protective factor contradicts the assertion in the SHAP analysis that its contribution is minimal. The authors need to either reconcile these findings with a clearer explanation or revise the interpretation to match the quantitative results. Additionally, the SHAP plots should be accompanied by more intuitive summaries, possibly including numerical summaries or stratified effects across risk groups.

Response: Thank you for this insightful comment. We agree that the initial interpretation of the SHAP results, particularly regarding education level, required refinement to ensure consistency with the quantitative findings.

In response, we have revised the SHAP-related interpretation throughout the manuscript to align more closely with the magnitude of the SHAP contributions. Specifically, while higher education level showed a directionally protective association, we now clarify that its relative predictive contribution was modest compared with pain-related features. Accordingly, education is no longer described as a dominant protective factor, but rather as a secondary contributor within the multivariable prediction framework (Discussion, page 16, paragraph lines 316–322).

In addition, to enhance interpretability beyond the SHAP plots, we have provided more intuitive summaries of the SHAP results. We added a narrative and numerical summary of feature importance and conducted a risk-stratified SHAP analysis, comparing key predictors across low-, moderate-, and high-risk groups. This stratified presentation highlights the heterogeneity of feature contributions across risk levels and facilitates a clearer understanding of the relative importance of pain-related symptoms, BMI, and education (Results, page 15, lines287–301).

We believe these revisions resolve the inconsistency noted by the reviewer and substantially improve the clarity and interpretability of the SHAP-based findings.

3.Comment 3:Third, the language of the manuscript requires modest revision to improve clarity and accuracy. While generally intelligible, the manuscript contains several lengthy and complex sentences that obscure meaning. These should be rewritten for conciseness and clarity. Furthermore, the authors should eliminate or rephrase any causal language, such as "mechanism," "regulatory role," or "explains the pathway," as the study is observational and not designed to infer causation. Appropriate phrasing should refer to "associations" or "predictive contributions."

Response:Thank you for this helpful comment. We agree that further refinement of the language was necessary to improve clarity, precision, and consistency with the observational nature of the study.

In response, we conducted a thorough language revision of the manuscript. Specifically, lengthy and complex sentences were rewritten to improve conciseness and readability, and ambiguous or potentially confusing phrasing was clarified throughout the text. In addition, all causal or mechanistic language—including terms such as “mechanism,” “regulatory role,” and “explains the pathway”—has been removed or rephrased. These expressions were replaced with terminology more appropriate for an observational and predictive study design, such as “association,” “predictive contribution,” or “risk pattern.”

These revisions were applied consistently across the Abstract, Results, and Discussion sections . We believe that these changes substantially improve the clarity of the manuscript and ensure that the interpretation of findings remains appropriately cautious and methodologically accurate.

4.Comment 4:Fourth, the description of the statistical pipeline could benefit from greater transparency. While the authors note the use of Bayesian optimization for hyperparameter tuning and stratified train/test splits, details on how the split was stratified (e.g., based on class distribution) and whether cross-validation was used for model robustness should be explicitly stated. Including a supplemental methods appendix with the full model training pipeline and Python/R code would enhance reproducibility.

Response:Thank you for this constructive comment. We agree that greater clarity regarding the statistical pipeline would improve transparency and reproducibility.

In response, we have substantially expanded the description of the analytical workflow in a newly added **Supplementary Methods** section. Specifically, we now explicitly state that the train/test split was performed using stratified sampling based on depression status to preserve class distribution, and that stratified cross-validation was applied during model training and hyperparameter tuning to enhance robustness under class imbalance. The use of Bayesian optimization, including its role in selecting optimal hyperparameters based on cross-validated performance, is also described in greater detail.

In addition, to further support transparency, we have included **Supplementary Code S1**, which provides an illustrative overview of the main analytical workflow (data preprocessing, feature selection, model training, hyperparameter tuning, and performance evaluation). This code is intended for illustrative purposes and does not represent a fully executable script, as some dataset-specific and environment-dependent components are omitted.

We believe that the combination of a detailed Supplementary Methods description and illustrative code sufficiently clarifies the statistical pipeline while remaining consistent with data governance requirements and the scope of the present study.

5.Comment 5:Fifth, while the data availability complies with PLOS ONE policies, it is recommended that the authors specify which CHARLS variables were used and provide their coding or labels in a supplementary table. This will support transparency for readers and future researchers aiming to replicate or extend the study.

Response:

Thank you for this helpful suggestion. We agree that explicitly documenting the variables used and their coding would further enhance transparency and facilitate reproducibility.

In response, we have added a new supplementary table summarizing all CHARLS variables included in the analysis, along with their corresponding labels, coding schemes, and brief descriptions. This table specifies how each variable was operationalized in the modeling process, including outcome definition, sociodemographic characteristics, pain-related variables.

The newly added table is provided as Supplementary Table 1, and it is referenced in the revised Methods section (Methods, page 6, lines124–125). We believe that this addition will assist readers and future researchers in replicating or extending the present analysis using CHARLS data, while remaining consistent with PLOS ONE data availability policies.

Response to Reviewer #2

1.Comment 1: .Please elaborate on the hyperparameter tuning process for each model. It is currently too vague.

Response:Thank you for this helpful comment. We agree that the description of the hyperparameter tuning process required further clarification.

In response, we have substantially expanded the description of hyperparameter optimization in the Methods section and Supplementary Methods. Specifically, hyperparameter tuning for all models was conducted using Bayesian optimization within the training dataset. For each model, a predefined search space was specified based on commonly recommended parameter ranges in the literature (e.g., regularization strength for logistic regression, kernel parameters for SVM, number of estimators and tree depth for ensemble models, and neighborhood size for KNN). Model performance during tuning was evaluated using stratified cross-validation to ensure robustness under class imbalance.

The optimal hyperparameter configurations were selected based on average cross-validation performance, rather than single-split results, to reduce the risk of overfitting. A concise summary of the tuning strategy and key hyperparameters for each model has now been explicitly described in the revised manuscript (Methods, page 6-7, lines 144–165; Supplementary Methods, page 22).

We believe that these revisions provide sufficient transparency regarding the hyperparameter tuning process while maintaining clarity and reproducibility of the analytical workflow.

2. Comment 2: Include ROC curves or AUC scores in addition to the reported metrics. This is a common practice in classification tasks.

Response: Thank you for this helpful suggestion. We agree that including ROC curves and AUC values provides a more comprehensive and threshold-independent evaluation of model performance.

In response, we have added ROC curves for all machine learning models and reported the corresponding AUC values in the revised manuscript. These metrics complement accuracy, precision, recall, and F1-score, and allow for a clearer comparison of the models’ discriminative ability, particularly under class imbalance. The ROC curves are presented in Figure 4, and the AUC values are summarized in Table 2 and described in the Results section (Results, page 12-13, lines 211–240).

We believe that the inclusion of ROC curves and AUC scores aligns the evaluation with common practice in classification studies and improves the interpretability and robustness of the model performance assessment.

3.Comment 3：The manuscript would benefit from restructuring the Discussion to more clearly separate interpretation from policy recommendations.

Response: Thank you for this valuable suggestion. We agree that a clearer separation between interpretation of findings and broader implications would improve the structure and readability of the Discussion.

In response, we have reorganized the Discussion section to more clearly distinguish between interpretation of the empirical results and their potential public health or policy implications. Specifically, we revised the early part of the Discussion to focus on interpreting the main findings in relation to existing literature and the predictive modeling framework, while methodological considerations and limitations are discussed separately. Broader implications are now presented in a distinct subsection and framed cautiously as potential implications rather than direct recommendations.

These revisions help ensure that interpretation remains grounded in the study’s observational and predictive nature, while policy-related considerations are clearly delineated and appropriately qualified (Discussion, page 16-18).

We believe that this restructuring improves conceptual clarity and aligns the Discussion more closely with the reviewer’s recommendation.

4.Comment 4：Add the sample size (n=38,970) to the Abstract for context.

Response: Thank you for this suggestion. We agree that explicitly reporting the sample size improves clarity and provides important context for the study.

Accordingly, we have added the sample size (n = 38,970) to the Abstract in the Methods section, where the study population is described (Abstract, page 1X, lines 22). We believe this revision enhances the transparency and readability of the Abstract.

5.Comment 5：The term “biphasic regulation” used for BMI should be briefly explained in lay terms.

Response:Thank you for this helpful suggestion. We agree that the term “biphasic regulation” may not be immediately clear to all readers and would benefit from a brief explanation in more accessible language.

In response, we have added a short clarification in the manuscript to explain that “biphasic regulation” refers to a non-linear, U-shaped association, whereby both relatively low and relatively high BMI values are associated with higher predicted depression risk, while intermediate BMI levels are associated with lower risk (Results, page 14, lines 259–265). This explanation is intended

Attachment Submitted filename: Response to Reviewers.docx

# Editor report: Decision Letter 1

**DOI:** 10.1371/journal.pone.0349135.r004

**Authors:** Naseer Muhammad Khan

26 Apr 2026

Exploration of comorbidity mechanisms between chronic pain and depression: Machine learning prediction models and SHAP interpretability analysis based on the CHARLS cohort

PONE-D-25-30274R1

Dear Dr. Liu,

We’re pleased to inform you that your manuscript has been judged scientifically suitable for publication and will be formally accepted for publication once it meets all outstanding technical requirements.

Within one week, you’ll receive an e-mail detailing the required amendments. When these have been addressed, you’ll receive a formal acceptance letter and your manuscript will be scheduled for publication.

An invoice will be generated when your article is formally accepted. Please note, if your institution has a publishing partnership with PLOS and your article meets the relevant criteria, all or part of your publication costs will be covered. Please make sure your user information is up-to-date by logging into Editorial Manager at Editorial Manager® and clicking the ‘Update My Information' link at the top of the page. For questions related to billing, please contact billing support.

If your institution or institutions have a press office, please notify them about your upcoming paper to help maximize its impact. If they’ll be preparing press materials, please inform our press team as soon as possible -- no later than 48 hours after receiving the formal acceptance. Your manuscript will remain under strict press embargo until 2 pm Eastern Time on the date of publication. For more information, please contact onepress@plos.org.

Kind regards,

Naseer Muhammad Khan, PhD

Academic Editor

PLOS One

Additional Editor Comments (optional):

Reviewers' comments:

# Editor report: Acceptance letter

**DOI:** 10.1371/journal.pone.0349135.r005

**Authors:** Naseer Muhammad Khan

PONE-D-25-30274R1

PLOS One

Dear Dr. Liu,

I'm pleased to inform you that your manuscript has been deemed suitable for publication in PLOS One. Congratulations! Your manuscript is now being handed over to our production team.

At this stage, our production department will prepare your paper for publication. This includes ensuring the following:

* All references, tables, and figures are properly cited

* All relevant supporting information is included in the manuscript submission,

* There are no issues that prevent the paper from being properly typeset

You will receive further instructions from the production team, including instructions on how to review your proof when it is ready. Please keep in mind that we are working through a large volume of accepted articles, so please give us a few days to review your paper and let you know the next and final steps.

Lastly, if your institution or institutions have a press office, please let them know about your upcoming paper now to help maximize its impact. If they'll be preparing press materials, please inform our press team within the next 48 hours. Your manuscript will remain under strict press embargo until 2 pm Eastern Time on the date of publication. For more information, please contact onepress@plos.org.

You will receive an invoice from PLOS for your publication fee after your manuscript has reached the completed accept phase. If you receive an email requesting payment before acceptance or for any other service, this may be a phishing scheme. Learn how to identify phishing emails and protect your accounts at https://explore.plos.org/phishing.

If we can help with anything else, please email us at customercare@plos.org.

Thank you for submitting your work to PLOS ONE and supporting open access.

Kind regards,

PLOS ONE Editorial Office Staff

on behalf of

Dr. Naseer Muhammad Khan

Academic Editor

PLOS One
